# Coupling metal and whole-cell catalysis to synthesize chiral alcohols

**DOI:** 10.1186/s40643-022-00560-0

**Published:** 2022-07-08

**Authors:** Hang Yin, Peng-Qian Luan, Yu-Fei Cao, Jun Ge, Wen-Yong Lou

**Affiliations:** 1 Lab of Applied Biocatalysis, School of Food Science and Engineering, South China University of Technology, No. 381 Wushan Road, Guangzhou, 510640 China; 2 Institute of Biomedical Health Technology and Engineering, Shenzhen Bay Laboratory, Shenzhen, 518132 China; 3 Key Lab for Industrial Biocatalysis, Ministry of Education, Department of Chemical Engineering, Tsinghua University, Beijing, China; 4 Institute of Biopharmaceutical and Health Engineering, Tsinghua Shenzhen International Graduate School, Shenzhen, 518055 China

**Keywords:** Metal catalysis, Whole-cell catalysis, Chemoenzymatic cascade catalysis, Chiral alcohol, (S)-4-chlorobenzhydrol

## Abstract

**Background:**

The combination of metal-catalyzed reactions and enzyme catalysis has been an essential tool for synthesizing chiral pharmaceutical intermediates in the field of drug synthesis. Metal catalysis commonly enables the highly efficient synthesis of molecular scaffolds under harsh organic conditions, whereas enzymes usually catalyze reactions in mild aqueous medium to obtain high selectivity. Since the incompatibility between metal and enzyme catalysis, there are limitations on the compatibility of reaction conditions that must be overcome.

**Findings:**

We report a chemoenzymatic cascade reaction involved Palladium (Pd) catalyzed Suzuki–Miyaura coupling and whole-cell catalyzed C = O asymmetric reduction for enantioselective synthesis of value-added chiral alcohol. The cell membrane serves as a natural barrier can protect intracellular enzymes from organic solvents.

**Conclusions:**

With dual advantages of cascade catalysis and biocompatibility, our work provides a rational strategy to harvest chiral alcohols in high yield and excellent enantioselectivity, as a channel to establish chemoenzymatic catalysis.

**Supplementary Information:**

The online version contains supplementary material available at 10.1186/s40643-022-00560-0.

## Introduction

With the characteristics of widely approved catalytic advantages, such as high selectivity, green and mild catalytic conditions, enzymes are widely used as a reliable tool to produce chemical compounds in the pharmaceutical industry (Cao et al. 2021; Wu et al. 2021). In contrast to enzyme catalysis, chemocatalysts are available to far more diverse types of reactions in high efficiency (Huang et al. 2020b). Given this, the integration of enzymes and chemocatalysts in a cascade pattern is drastically explored for the combination of the excellent selectivity of the former with the robust reactivity of the latter (Huang et al. 2020a). In addition, the hurdle of having to isolate intermediates in the combination of enzymes and chemocatalysts can be avoided. Hence many research works on integrating enzymes with chemocatalysts have been reported (Maaskant et al. 2021; Mathew et al. 2021). Although widely applied in pharmaceutical manufacturing industry, the cascade systems also face a major challenge because of the incompatibility in catalytic reactions (Cao and Ge 2021). In general, enzymes cannot tolerate the harsh condition, such as organic solvents, acidic or alkaline conditions and high temperatures that chemocatalysts are prone to perform optimally (Ni et al. 2021). To fill in this gap between chemocatalysts and enzymes, substantial scientific efforts have been channeled to resolve this impasse (Rudroff et al. 2018; Schmidt et al. 2018).

(S)-4-chlorobenzhydrol is a type of important chiral pharmaceutical intermediates, the efficient synthesis route of which has been always pursuit to achieve high yield and high selectivity (Bolm and Rudolph 2002). Touge et al. prepared chiral benzhydrols via asymmetric transfer hydrogenation of unsymmetrical benzophenones with costly bifunctional oxo-tethered ruthenium catalysts (Touge et al. 2016). However, 4-chlorobenzhydrol was formed with only a moderate enantiomeric excess (ee) of 48%. Compared with metal catalysts, a novel ketoreductase KmCR2 can catalyze diarylmethanone substrates to obtain diarylmethanols in high enantioselectivity (Li et al. 2019). Most recently, Manna et al. reported amino acid-functionalized metal–organic frameworks for asymmetric base-metal catalysis, with the catalyst affording a high yield (99%) and enantioselective (99%) for the successful preparation of 4-chlorobenzhydrol (Newar et al. 2021). Unfortunately, the process cannot directly convert ketone substrates to chiral alcohols, which requires an additional hydrolytic step. Furthermore, the reaction is typically carried out in the presence of THF, with the wastes produced being notoriously toxic and harmful. Still, Bruce’s group reported a surfactant, TPGS-750-M, which forms nanomicelles in water and enables an one-pot cascade reaction involving both chemo- and bio-conversions (Cortes-Clerget et al. 2019). Moreover, Lee et al. synthesized a round bottom jar-like silica nanostructure to load enzyme and chemocatalyst for the cascade catalysis (Kim et al. 2021).

**Scheme 1:**
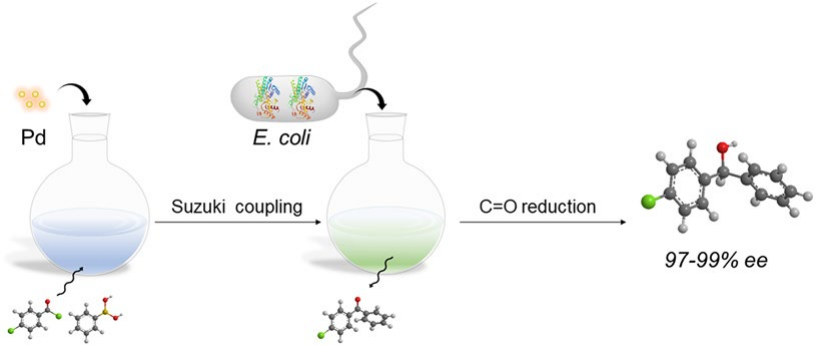
Schematic diagram of one pot chemoenzymatic process for (S)-4-chlorobenzhydrol

Compared with enzymes, the whole cell catalysts can serve as a natural immobilized enzyme. The cell membrane provides natural protection for intracellular enzymes. Furthermore, the purification of enzymes from the whole-cells is also avoided. The chemoenzymatic catalysis combining the wide catalytic capabilities of chemocatalysts and the exquisite selective properties of whole-cell biocatalysts, is considered as an effective tool for developing novel and functional reactions (Huang et al. 2015). Therefore, in this study, we report a chemoenzymatic approach towards the synthesis of 4-chlorobenzhydrol, which combining Pd-catalyzed C–C formation and whole cell-catalyzed C=O reduction. We achieved the direct enantioselective synthesis of (S)-4-chlorobenzhydrol in high yield and excellent enantioselectivity from readily available phenylboronic acid and 4-chlorobenzoyl chloride as substrates (Scheme 1). We demonstrate the possibility of coupling of whole-cell catalysis and metal catalysis. This composite catalyst could be used to synthesize a type of chiral alcohol products. In this process, the aryl groups were incorporated through Suzuki–Miyaura coupling (SMC) catalyzed by tetrakis(triphenylphosphine)palladium, and subsequently, the stereocenter was generated by whole-cell catalyzed reductive reactions.

## Results and discussion

We started from investigating Pd-catalysis and whole-cell catalysis separately. To identify a suitable Pd catalyst, we selected three commercial catalysts Pd(PPh_3_)_4_, Pd(OAc)_2_ and Pd/C (Table 1, entries 1–3). At the outset, to evaluate the performance of the Pd catalyst, the reaction of 4-chlorobenzoyl chloride (0.11 mmol), phenylboronic acid (0.1 mmol), Cs_2_CO_3_ (1 mmol) and catalyst (5 mol%) was chosen as the initial model and conducted in toluene at 80 °C. As the results shown in Table 1, the homogeneous catalyst, Pd(PPh_3_)_4,_ reaches an excellent yield of 76% within 30 min. It should be noticed that the yield of product was determined using HPLC. Subsequently, the effect of different solvents and bases were tested. The results suggested that the addition of K_2_CO_3_ and the use of toluene can lead to a better yield of up to 89% of 4-chlorobenzophenone (Table 1, entries 4–10). In view of the biocompatibility of co-solvent, toluene was replaced by xylene as reaction solvent, the yield of ketone appeared only slightly less than the yield under solvent of toluene (Table 1, entry 7). Besides, the para-position of chlorine atom scarcely performs reactivity in the coupling reaction with phenylboronic acid in xylene, as monitored by GC–MS analysis (Additional file 1: Fig. S5). Therefore, we could mainly harvest required products of ketone rather than by-products.

**Table 1:** Optimization of reaction conditions for screening catalyst and base


Entry	Pd catalyst	Solvent	Base	Temperature (°C)	Time (h)	Yield (%)
1	Pd(OAc)_2_	Toluene	Cs_2_CO_3_	80	0.5	12
2	Pd/C	Toluene	Cs_2_CO_3_	80	0.5	7
3	Pd(PPh_3_)_4_	Toluene	Cs_2_CO_3_	80	0.5	76
4	Pd(PPh_3_)_4_	MeOH	Cs_2_CO_3_	80	0.5	25
5	Pd(PPh_3_)_4_	H_2_O	Cs_2_CO_3_	80	0.5	9
6	Pd(PPh_3_)_4_	2-MeTHF	Cs_2_CO_3_	80	0.5	61
7	Pd(PPh_3_)_4_	Xylene	Cs_2_CO_3_	80	0.5	72
8	Pd(PPh_3_)_4_	Toluene	K_2_CO_3_	80	0.5	89
9	Pd(PPh_3_)_4_	Toluene	LiOH·H_2_O	80	0.5	57
10	Pd(PPh_3_)_4_	Toluene	TEA	80	0.5	64
11	Pd(PPh_3_)_4_	Xylene	K_2_CO_3_	80	3	98
12	Pd(PPh_3_)_4_	Xylene	K_2_CO_3_	25	3	10
13	Pd(PPh_3_)_4_	Xylene	K_2_CO_3_	60	3	70
14	Pd(PPh_3_)_4_	Xylene	K_2_CO_3_	70	3	77
15	Pd(PPh_3_)_4_	Xylene	K_2_CO_3_	90	3	97

**Fig. 1:**
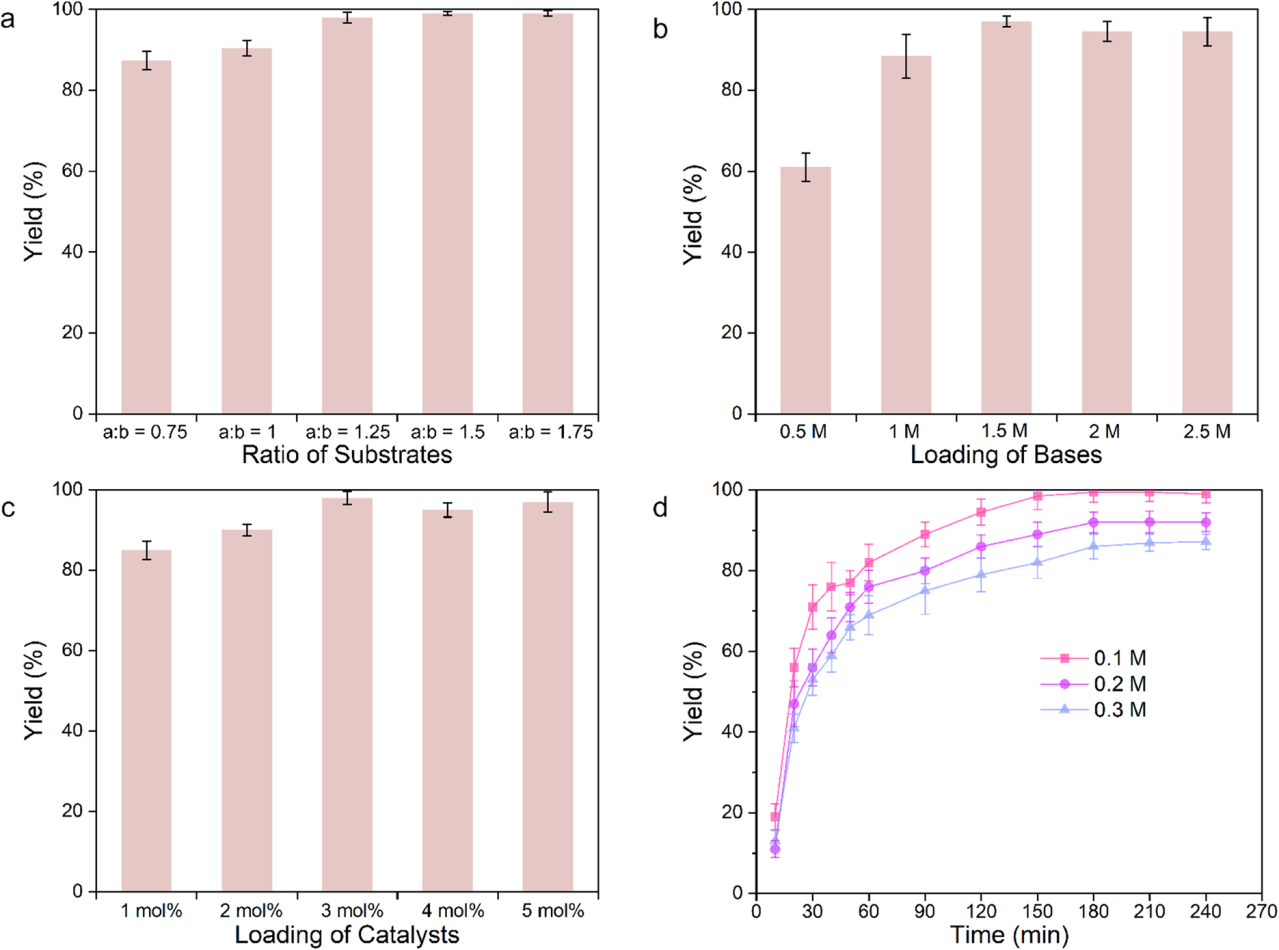
Optimization studies of Pd catalysis. (**a**) Ratio of substrates (a: 4-chlorobenzoyl chloride; b: phenylboronic acid); (**b**) loading of bases; (**c**) loading of catalysts; (**d**) reaction time and different concentrations of substrates of 4-chlorobenzoyl chloride

We then performed the reactions in the presence of various reactant ratios. From Fig. 1a, we found the best yield of 98% was harvested when the ratio between 4-chlorobenzoyl chloride and phenylboronic acid was 1.5. For the optimization of the loadings of catalyst and base (Fig. 1b, c), the results indicated that 3 mol% catalyst and 1.5 M base led to a high yield of 4-chlorobenzophenone.The Suzuki-coupling reaction of three different quantities of ketones was evaluated under the aforementioned optimized conditions. As shown in Fig. 1d, the yield of ketone can reach 80% in 1 h when catalyzed by Pd(PPh_3_)_4_ in the concentration of 0.1 mM 4-chlorobenzoyl chloride. The yield of reaction reached 99% within followed 2 h. We observed a similar trend with different concentrations of 4-chlorobenzoyl chloride.

**Fig. 2:**
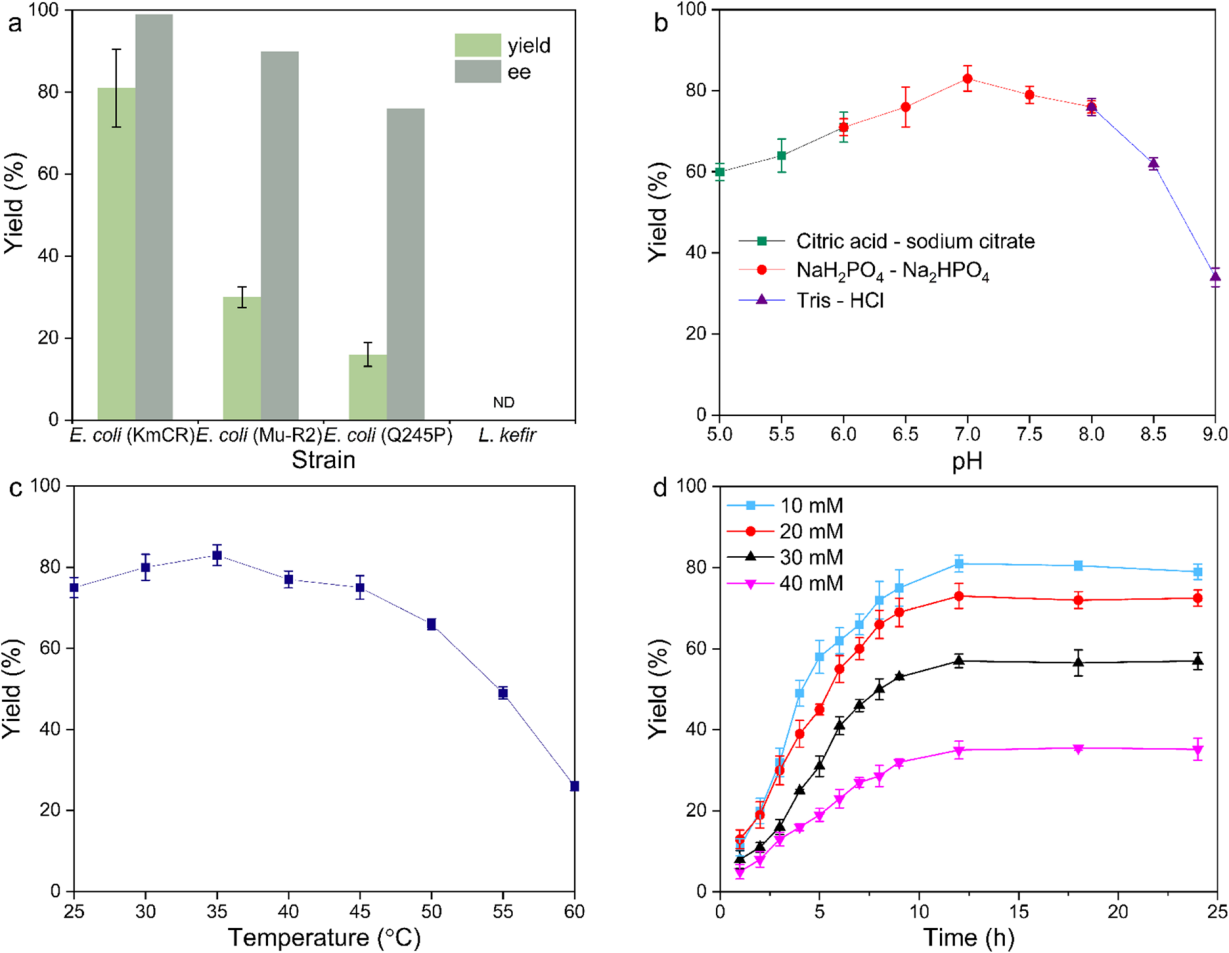
Comparison of different strains and optimization concentrations of E. coli (KmCR). (**a**) Strains; (**b**) pH; (**c**) reaction temperature; (**d**) reaction time

After the optimization of the Pd-catalyzed synthesis of 4-chlorobenzophenone, we focused on the second step of cascade reaction, i.e., the whole-cell catalyzed reduction of the diaryl ketone to chiral alcohol. We chose three engineering bacteria and one wild strain as alternative to investigate the best option with appealing enantioselective yield of (S)-4-chlorobenzhydrol. Compared with three other strains, the E. coli–KmCR mutant performed a superior yield and selectivity (Fig. 2a). Followed by the optimization of pH and temperature, we could obtain the alcohol in 81% yield and 99% ee (S) (Fig. 2b, c). Furthermore, different quantities of substrates were added to assess the catalysis performance of the whole-cell catalyst. As shown in Fig. 2d, within the first 6 h, 4-chlorobenzhydrol had accumulated at a yield of 60%, indicating that the 4-chlorobenzophenone was a suitable substrate for the whole-cell reduction. Thereafter, the catalysis slowed down after 12 h. Besides, with the increase of the substrates concentration, the product yield was decreased, which might be due to the toxicity of high-concentration substrates.

**Fig. 3:**
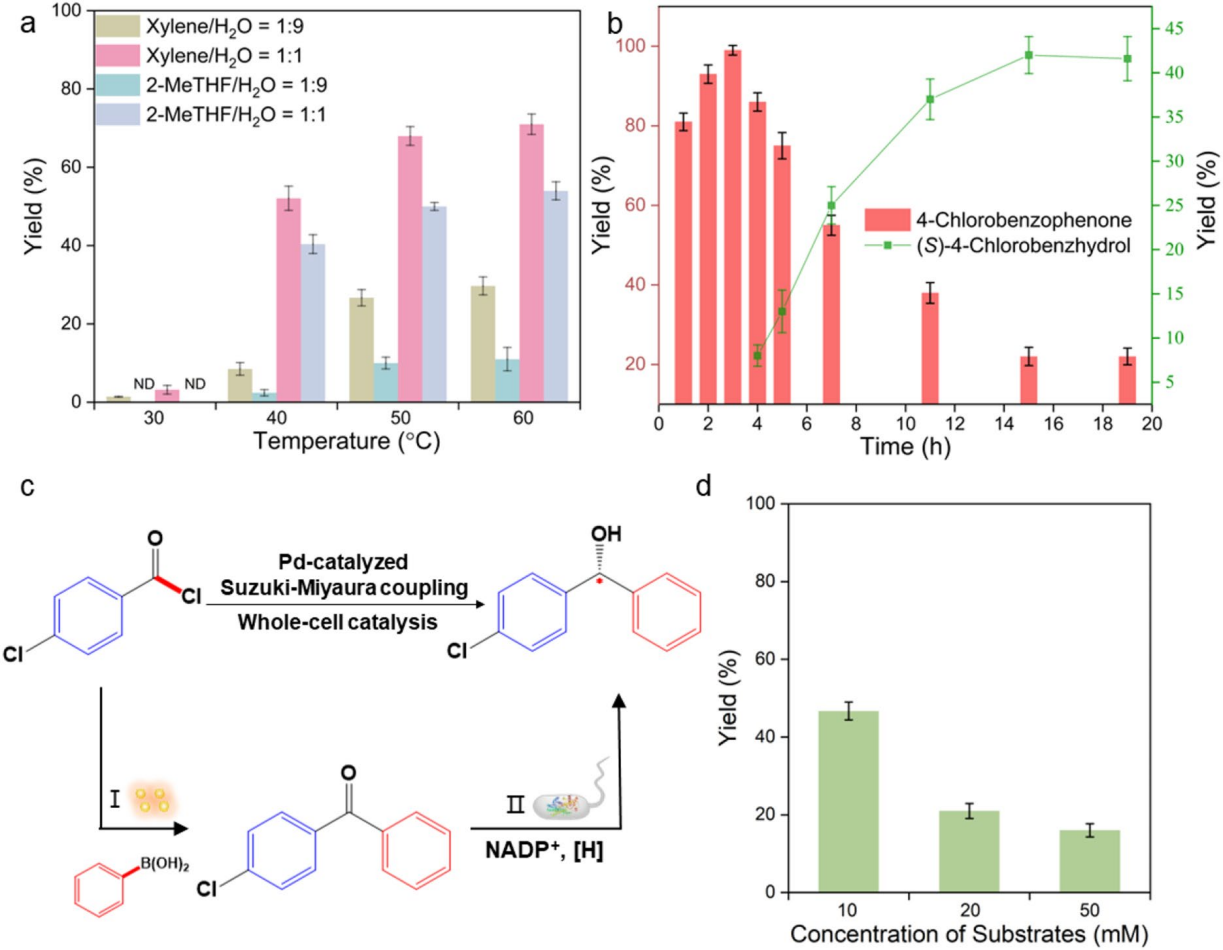
Chemo-enzymatic cascade catalysis. (**a**) Yield of ketone in the organic–aqueous system; (**b**) yield of alcohol at the one-pot sequential process; (**c**) sequential reaction diagram; (**d**) yield of alcohol at different concentrations of substrates (4-chlorobenzoyl chloride)

Then we tried to combine the Pd-catalysis and whole-cell catalysis in an one-pot tandem chemoenzymatic process, which is attractive for its easy-operating procedure (Wu et al. 2019). To address the challenge of incompatibility between metal catalysis and biocatalysis, a two-phase solvent (organic–aqueous) system was applied. As shown in Fig. 3a, two different organic solvents were tested, involving hydrophobic xylene and hydrophilic 2-MeTHF. We found that the water content of the two-phase systems can directly affect the yield of ketone. Furthermore, as the temperature increased, the yield of ketone correspondingly rose. Notably, when the temperature was 40 ℃, 50 ℃ and 60 ℃, the yield of ketone can reach more than 50%. Then we tested whole-cell catalysis in the same conditions. From Additional file 1: Fig. S2a, it indicated that the yield of whole-cell catalysis can reach 50% at 40 ℃ and 50 ℃ in the two-phase system of xylene/PB in a ratio of 1:1. Since each single catalyst (both Pd and whole-cell catalyst) can harvest products with reasonable yields, we, therefore, explored the one-pot reaction in the two-phase system at 40 ℃ and 50 ℃, involving Pd catalyst and the whole-cell catalyst in the same reaction unit. Unexpectedly, we just detected the formation of 4-chlorobenzophenone, the product of the Pd catalysis, but the final product, the desired alcohol was not formed (Additional file 1: Fig. S2b). This indicated that whole-cell catalyst may lose its corresponding activity when the Pd catalyst was simultaneously running. To investigate the reason of low activity of whole-cell catalyst in the one-pot process, we evaluated the effect of each individual component in the one-pot process on the activity of whole-cell catalyst. In addition, we found that every component, including 4-chlorobenzoyl chloride, phenylboronic acid and K_2_CO_3_ and Pd(PPh_3_)_4_ reduced the activity of whole-cell catalyst to some extent (Additional file 1: Fig. S3). In the SEM image of Additional file 1: Fig. S4, it observed that some broken structures of the cell wall in E.coli were appeared after the one-pot catalytic process. This indicated that several components applied in the one-pot process caused the deactivation of E.coli.

Therefore, to establish the Pd-whole cell cascades, we finally carried out a sequential chemoenzymatic mode which could avoid the deactivation of whole-cell catalyst. Having, respectively, tested the optimal reaction conditions of Pd-catalysis and whole-cell catalysis, we targeted a stepwise process (Fig. 3c) which can display maximum specific activity without limiting individual catalysis. In the first stage, Pd catalysis was carried out in 3 mol% and within 3 h ketone intermediate gathered in 99% yield (Fig. 3b). Then PB buffer was mixed with the organic solvent, and pH was adjusted to 7, followed by the addition of whole-cell catalyst and cofactors. Subsequently, the whole-cell catalyst started to convert ketone to enantioenriched alcohol. Finally, we could harvest (S)-4-chlorobenzhydrol in 42% yield. We also implemented higher concentrations of substrate and obtained more products. Compared to 10 mM 4-chlorobenzoyl chloride, the use of 50 mM substrate led to a less yield (Fig. 3d).

## Conclusions

In summary, we developed a metal–whole cell cascade process that enables the conversion of acyl chloride to enantioenriched alcohols. To overcome the inherent challenges between chemocatalytic and biocatalytic process, we adopted a sequential strategy involving the Pd-catalyzed Suzuki–Miyaura coupling of acyl chloride and the whole-cell reduction of diaryl ketone intermediates. This effort of metal–whole cell catalysis integrates advantages from both types of catalysts and will pave the way for efficient synthesis of diverse chiral chemical compounds.

### Supplementary Information

**Additional file 1: Figure S1.** (a) HPLC calibration of 4-chlorobenzophenone at 254 nm. (b) HPLC calibration of (*S*)-4-chlorobenzhydrol at 210 nm. **Figure S2.** (a) Whole-cell catalyzed reaction at two-phase solvent. (b) Red column: yield to ketone of a tandem catalytic system, ND means no detected of alcohol. This means only ketone detected in concurrent catalytic system. **Figure S3.** Effect of different single addition of reaction component to whole cell catalysis (PA: phenylboronic acid; pCC: 4-chlorobenzoyl chloride). **Figure S4.** (A), (B) SEM of *E. coli*. (C), (D): *E. coli* stature after chemoenzymatic reaction. **Figure S5.** GC–MS analysis of 4-chlorobenzophenone. **Figure S6.** HPLC analysis of 4-chlorobenzophenone and (*S*)-4-chlorobenzhydrol. **Figure S7.** ^1^H NMR and ^13^C NMR spectrum for 4-chlorobenzophenone.

## Data Availability

All data generated and analysed during this study are included in this published article and its supplementary information file.

## Abbreviations

THF: Tetrahydrofuran
2-MeTHF: 2-Methyltetrahydrofuran
NADPH: Nicotinamide adenine dinucleotide phosphate
GC–MS: Gas chromatography–mass spectrometry
