# Supplementary material for: Coupling metal and whole-cell catalysis to synthesize chiral alcohols
Source: Bioresour Bioprocess. 2022 Jul 8;9(1):73. doi: 10.1186/s40643-022-00560-0 (PMC10992956; doi:10.1186/s40643-022-00560-0)
Supplement: Supplementary file 1 — Additional file 1: Figure S1. (a) HPLC calibration of 4-chlorobenzophenone at 254 nm. (b) HPLC calibration of (S)-4-chlorobenzhydrol at 210 nm. Figure S2. (a) Whole-cell catalyzed reaction at two-phase solvent. (b) Red column: yield to ketone of a tandem catalytic system, ND means no detected of alcohol. This means only ketone detected in concurrent catalytic system. Figure S3. Effect of different single addition of reaction component to whole cell catalysis (PA: phenylboronic acid; pCC: 4-chlorobenzoyl chloride). Figure S4. (A), (B) SEM of E. coli. (C), (D): E. coli stature after chemoenzymatic reaction. Figure S5. GC–MS analysis of 4-chlorobenzophenone. Figure S6. HPLC analysis of 4-chlorobenzophenone and (S)-4-chlorobenzhydrol. Figure S7. 1H NMR and 13C NMR spectrum for 4-chlorobenzophenone. [file 40643_2022_560_MOESM1_ESM.docx]

Additional information for

**Coupling Metal and Whole-Cell Catalysis to Synthesize Chiral Alcohols**

Hang Yin ^1,2,†^, Peng-Qian Luan ^2,†^, Yu-Fei Cao ^3^, Jun Ge ^3,4,2,*^, Wen-Yong Lou ^1,*^

^*^ Corresponding author. E-mail: [junge@mail.tsinghua.edu.cn](mailto:junge@mail.tsinghua.edu.cn); [wylou@scut.edu.cn](mailto:wylou@scut.edu.cn)

^†^ Hang Yin and Peng-Qian Luan contributed equally to this work

^1^ *Lab of Applied Biocatalysis, School of Food Science and Engineering, South China University of Technology, No. 381 Wushan Road, Guangzhou 510640, China*

^2^ *Institute of Biomedical Health Technology and Engineering, Shenzhen Bay Laboratory, Shenzhen 518132, China*

^3^ *Key Lab for Industrial Biocatalysis, Ministry of Education, Department of Chemical Engineering, Tsinghua University, Beijing, China*

^4^ *Institute of Biopharmaceutical and Health Engineering, Tsinghua Shenzhen International Graduate School, Shenzhen 518055, China*

**Experimental section**

***Materials:*** The ketone starting materials: 4-chlorobenzoyl chloride and phenylboronic acid were purchased from Acros Organics. Pd catalysts and some commercially available chemicals were purchased from Sigma-Aldrich. The standard samples of Chiral alcohol were obtained from Bide Pharmatech Ltd. and synthesized from Pharmacore Ltd. *E*.*coli* (KmCR) and *E*.*coli* (Q245P) were donated from Fudan University and Tianjin Institute of Industrial Biotechnology, Chinese Academy of Sciences. *E*.*coli* (Mu-R2) was constructed by BGI Genomics Ltd. according to the method described in a previous study (Zhou et al. 2018). *L. kefir* was obtained from China General Microbiological Culture Collection Center (CGMCC). Deionized water was purified using a Q-POD Ultrapure Water Remote Dispenser from Millipore equipped with a Millipak Express 40 Q-POD (0.22 µm filter). Isopropyl alcohol HPLC grade and n-Hexane was obtained from Millipore Sigma. Determination of yield and enantiopurity was carried out on Agilent HPLC 1260 II using Daicel Chiral AD-H column. Products were confirmed by 5977B GC/MSD and ^1^H NMR and ^13^C NMR spectral data with reported literature. ^1^H NMR and ^13^C NMR spectra were recorded using 400 MHz with samples in DMSO-d6 solutions at 25 ℃ with a Bruker ;AVANCE III 400 (Rafiee and Hajipour 2015). Bacteria were characterized by scanning electron microscopy (SEM) analysis (ZEISS SUPRA^®^ 55).

***Palladium-catalyzed Suzuki-Miyaura reaction of acyl chloride and phenylboronic acid:*** In a typical experiment, a 10 mL vial containing a magnetic stir was charged with 4-chlorobenzoyl chloride (0.11 mmol), Phenylboronic acid (0.1 mmol), Cs_2_CO_3_ (0.1 mmol) and Pd[P(C_6_H_5_)_3_]_4_ (5mol%). Subsequently, the vial was added 1 mL toluene and stirred at 80 ℃ for 3 h. After cooling to 25 ℃, the substances in vial were purified through a 13 mm × 0.22 µm nylon filter. The products were analyzed using GC-MS and NMR (Dander et al. 2019). The ketone yields were calculated using HPLC.

***Whole-cell catalyzed reduction to produce (S)-4-chlorobenzhydrol:*** To 10 mM ketone substrate, 20 mM glucose, 1 mg NADP^+^ in 200 mM PB buffer (pH 7.0) were added 0.2 g wet cell and 1mg glucose dehydrogenase (GDH). A 20 mL vial containing 5 mL of the above mixture was shaken with a magnetic stir at 500 rpm and 35 ℃. After 12 h, the reaction mixture was extracted with EtOAc (5 mL × 3), then the organic layer was extracted, dried over Na_2_SO_4_ and analyzed by GC-MS. The product was confirmed using GC-MS on Agilent 5977B equipped with HP-5MS column (30 m × 0.25 mm, 0.25 mm film). The temperature program is as follows: 80 ℃ for 3 min, 30 ℃/min from 80 to 280 ℃, 280 ℃ for 5 min. The ee of the products were analyzed using HPLC. The characterization data from chiral HPLC analysis were Chiracel^®^ AD-H, 250 × 4.6 mm column, hexane/2-propanol 90:10, 0.7 mL/min flow rate, 254 nm and 210 nm UV lamp.

***Screening of optimal strain to produce (S)-4-chlorobenzhydrol:*** *E. coli* **(**KmCR), *E. coli* (Mu-R2), *E. coli* (Q245P) and *L.kefir* were cultured according to the previous reports. The collected bacteria were stored at 4 ℃. To a 20 mL reaction vial, 10 mM 4-chlorobenzophenone, 20 mM glucose, 1 mg NADP^+^ in 5 mL 200 mM PB (pH 7.0) were added 0.2 g wet cell and 1mg GDH. The vial was shaken with a magnetic stir at 500 rpm and 35 ℃. After 12 h, the reaction mixture was extracted with EtOAc (5 mL × 3), then the organic layer was extracted, dried over Na_2_SO_4_ and analyzed by HPLC. The characterization data from chiral HPLC analysis were Chiracel^®^ AD-H, 250 × 4.6 mm column, hexane/2-propanol 90:10, 0.7 mL/min flow rate, 254 nm and 210 nm UV lamp.

***Tandem reaction:*** Freshly harvested cells and Pd catalysts were resuspended in a 5 mL two phase solvent mixed 200 mM PB buffer with xylene. To the reaction vial, 20 mM glucose, 1 mg NADP^+^ and 1 mg GDH were added to serve the catalytic system. The reaction was reacted at 500 rpm, 50 ℃ for 12 h. After cooling to 25 ℃, EtOAc(5 mL × 3) was added. Then organic layer was dried over Na_2_SO_4_ and analyzed by GC-MS and HPLC.

***One-pot sequential cascade reaction****:* To a 20 mL vial, 4-chlorobenzoyl chloride (0.11 mmol), phenylboronic acid (0.1 mmol), Cs_2_CO_3_ (0.1 mmol) and Pd[P(C_6_H_5_)_3_]_4_ (5mol%) were added. Then 1 mL xylene were added with a magnetic stir. The reaction was performed at 80 ℃ for 3 h. After the first step reaction, the temperature of reaction should be down to 25 ℃. Then the 4 mL 200 mM PB buffer was added. The two-phase solvents could be shaken with a magnetic stir for 5 minutes. The pH was adjusted to 7 with HCl solution (0.5M). Then 0.2 g wet cell was added with 20 mM glucose, 1 mg NADP^+^ and 1mg GDH. The reaction was performed at 35 ℃ for 12 h. The reaction was dissolved in EtOAc (5 mL × 3). The organic layer was dried over Na_2_SO_4_.The product was determined by HPLC and GC-MS.


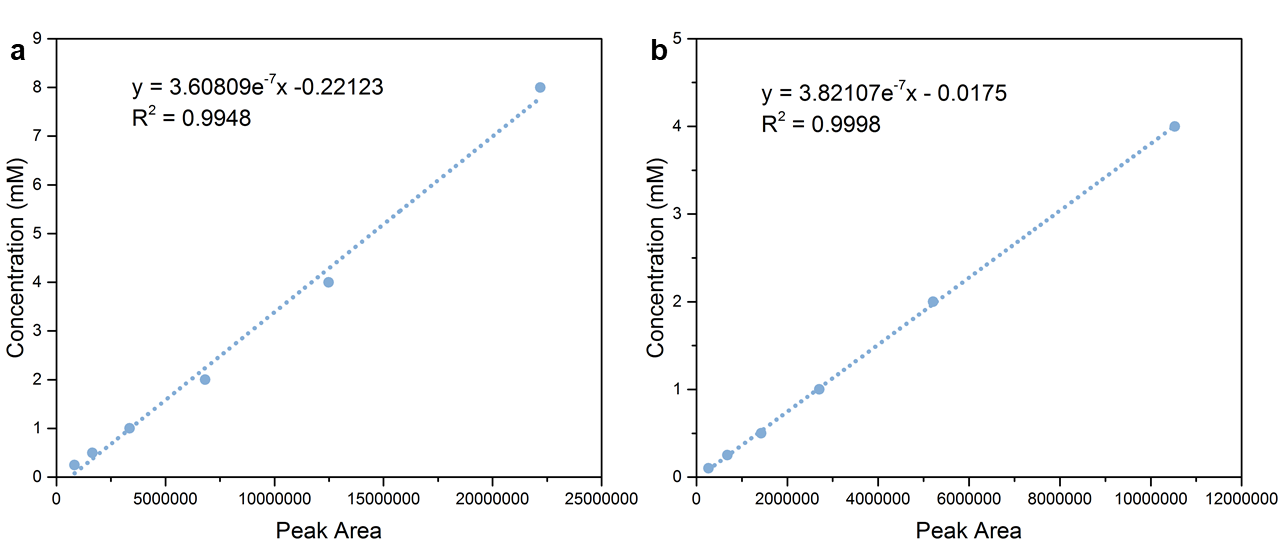


**Fig. S1.** (a) HPLC calibration of 4-chlorobenzophenone at 254 nm. (b) HPLC calibration of (*S*)-4-chlorobenzhydrol at 210 nm.


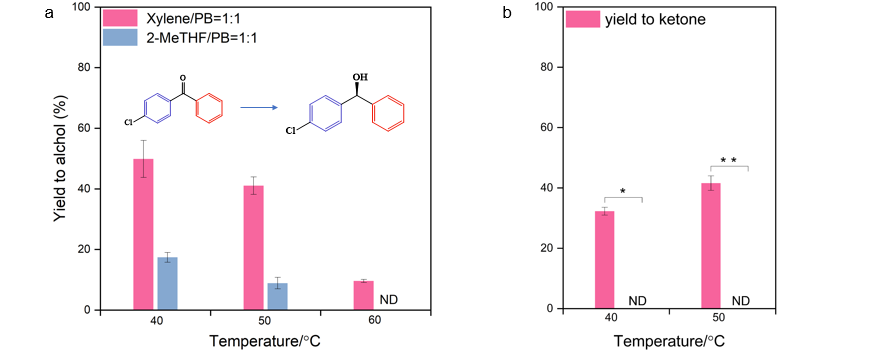


**Fig. S2.** (a) Whole-cell catalyzed reaction at two-phase solvent. (b) Red column: yield to ketone of a tandem catalytic system, ND means no detected of alcohol. This means only ketone detected in concurrent catalytic system.


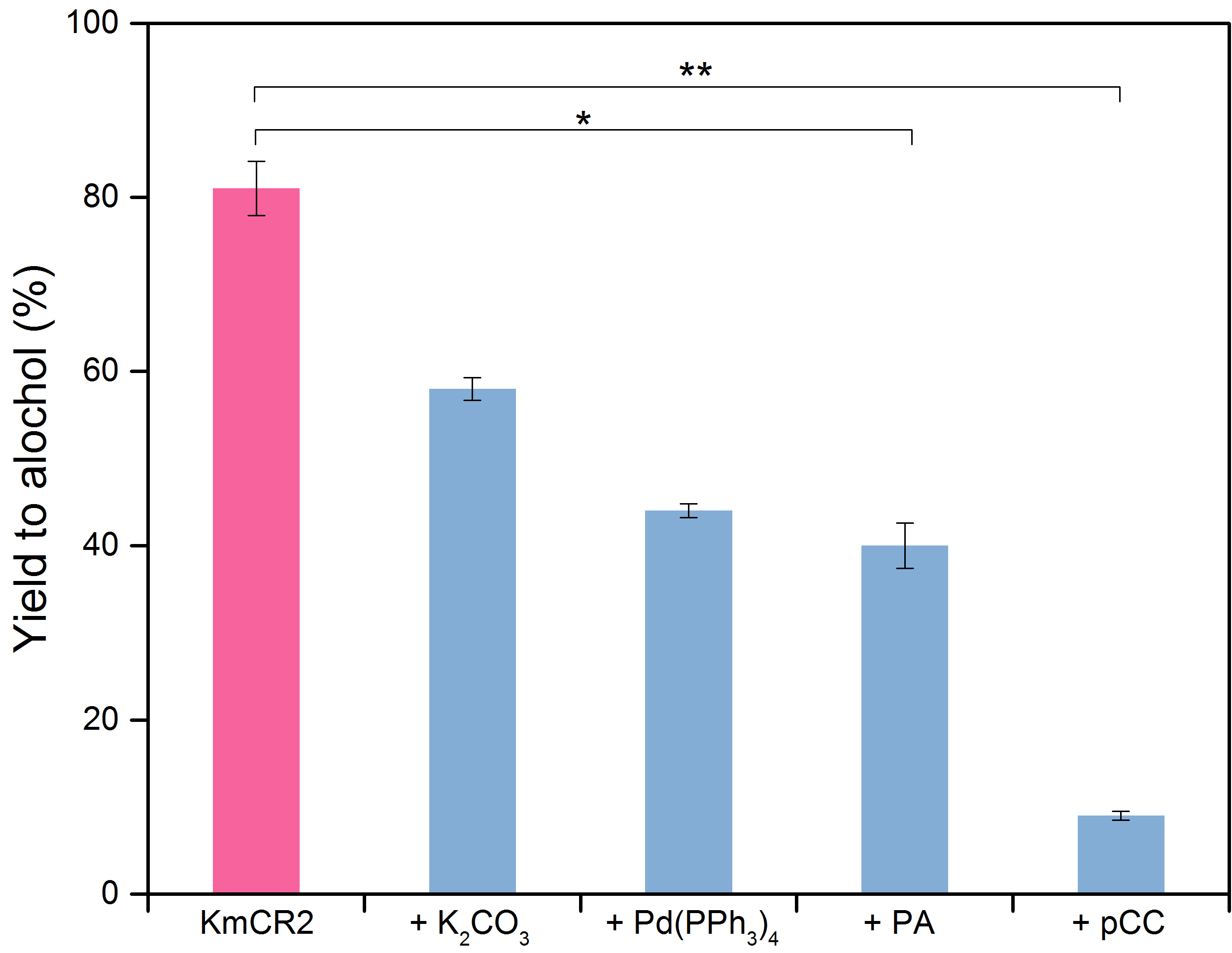


**Fig. S3.** Effect of different single addition of reaction component to whole cell catalysis (PA: phenylboronic acid; pCC: 4-chlorobenzoyl chloride).


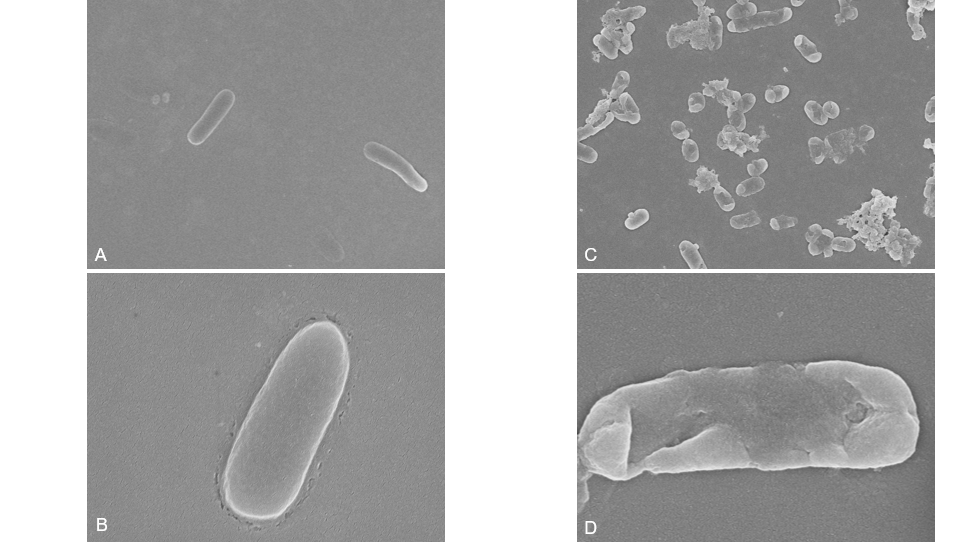


**Fig. S4.** (A), (B) SEM of *E. coli*. (C), (D): *E. coli* stature after chemoenzymatic reaction.


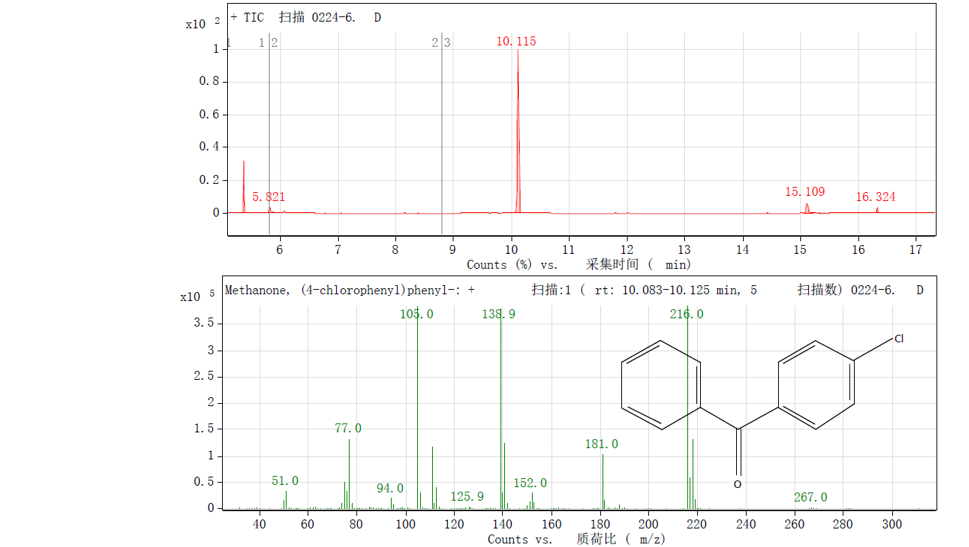


**Fig. S5.** GC-MS analysis of 4-chlorobenzophenone.


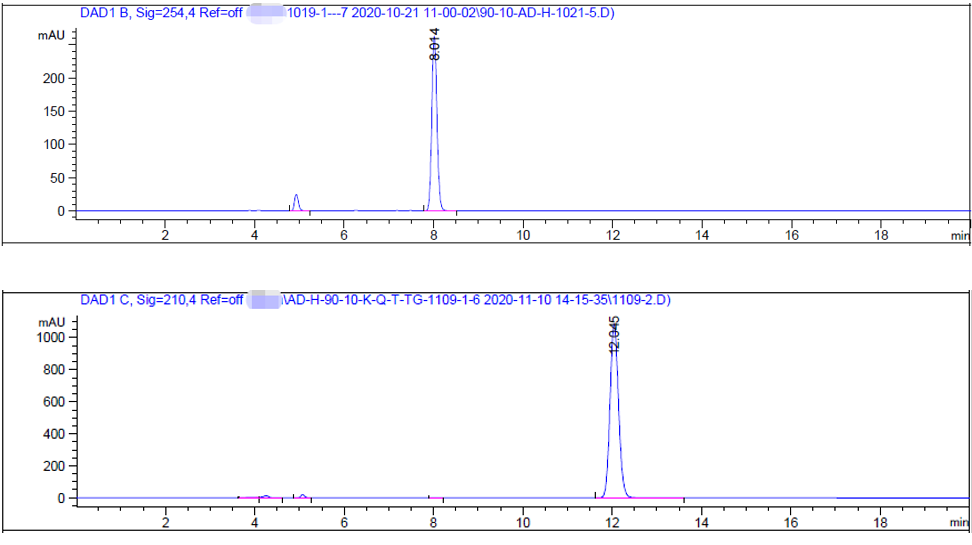


**Fig. S6.** HPLC analysis of 4-chlorobenzophenone and (*S*)-4-chlorobenzhydrol.

**^1^H NMR** (400 MHz, DMSO-*d*_6_) δ 7.89 – 7.46 (m, 9H). **^13^C NMR** (101 MHz, DMSO) δ 195.19, 138.09, 137.16, 136.18, 133.36, 131.96, 130.06, 129.20, 129.12.

**Fig. S7.** ^1^H NMR and ^13^C NMR spectrum for 4-chlorobenzophenone.

**^1^H NMR** (400 MHz, DMSO-d6) δ 7.53-7.10 (m, 9H), 5.97 (d, *J* = 3.9 Hz, 1H), 5.71 (d, *J* = 3.8 Hz, 1H). **^13^C NMR** (101 MHz, DMSO) δ 145.73, 145.17, 131.67, 128.62, 128.52, 128.49, 127.35, 126.69, 73.96.

**Fig. S8.** ^1^H NMR and ^13^C NMR spectrum for (*S*)-4-chlorobenzhydrol.

**Reference**

Dander JE, Giroud M, Racine S, Darzi ER, Alvizo O, Entwistle D, Garg NK (2019) Chemoenzymatic conversion of amides to enantioenriched alcohols in aqueous medium. Comm Chem 2(1):82 <https://doi.org/10.1038/s42004-019-0182-8>

Rafiee F, Hajipour AR (2015) A versatile method for the synthesis of diaryl and alkyl aryl ketones via palladium-catalysed cross-coupling reaction of arylboronic acids with acyl chlorides. Appl Organomet Chem 29(3):181-184 <https://doi.org/10.1002/aoc.3269>

Zhou J, Wang Y, Xu G, Wu L, Han R, Schwaneberg U, Rao Y, Zhao Y-L, Zhou J, Ni Y (2018) Structural Insight into Enantioselective Inversion of an Alcohol Dehydrogenase Reveals a “Polar Gate” in Stereorecognition of Diaryl Ketones. J Am Chem Soc 140(39):12645-12654 <https://doi.org/10.1021/jacs.8b08640>
